# Non-lethal suicidal behavior in university students of Spain during COVID-19

**DOI:** 10.3389/fpsyt.2023.1155171

**Published:** 2023-07-18

**Authors:** Javier Ramos-Martín, José Manuel Pérez-Berlanga, Jesús Oliver, Berta Moreno-Küstner

**Affiliations:** ^1^Departamento de Personalidad, Evaluación y Tratamiento Psicológico, Universidad de Málaga, Málaga, Spain; ^2^Grupo Andaluz de Investigación Psicosocial (GAP) (CTS-945), Málaga, Spain; ^3^Departamento de Psicología, Facultad de Ciencias Humanas y Sociales, Universidad Pontificia Comillas, Madrid, Spain; ^4^Instituto de Biomedicina de Málaga (IBIMA), Málaga, Spain

**Keywords:** suicidal behavior, mental health, prevalence, college students, COVID-19 pandemic

## Abstract

**Background:**

Suicide is the fourth external cause of death in the world, in persons between the ages of l5 and 29. The objectives of this study were to measure the prevalence of suicidal behavior in university students and analyze the relationship of suicide risk with psychological distress, resilience, and family and social support.

**Methods:**

An observational and transversal study wherein the students at the University of Malaga (Spain) completed an online questionnaire which included items from different scales, sociodemographic and academic questions, and the subjective impact of the COVID-19 pandemic. Descriptive analyses and prevalence rates of suicidal behavior were calculated, and bivariate analyses, multiple linear regression, and a mediation and moderation analyses were conducted.

**Results:**

A total of 2,212 students completed the questionnaire. The prevalence of the last 6 months was 30.4% wishing for death, 14.7% suicidal ideation, 5% self-harm injuries, and 0.5% suicide attempts. Psychological distress, family and social support were linked to the risk of suicide. Lastly, resilience and family support measure and moderate the relation between psychological stress and suicide risk.

**Conclusion:**

Psychological distress is a risk factor for suicidal behavior, while resilience and family and social support are linked to a lower risk of suicide.

## Background

Completed suicide may appear at any age and within the 15 to 29-years-old age group, in Spain, it presents itself with the lowest rate of suicide compared with other age groups (1). Despite this, suicide is the fourth external cause of death in people between 15 and 29 years in the world (2).

University students make up a vulnerable group with the appearance of mental illnesses (3–5). Among the most common during the university stage are depression, anxiety and disorders through substance use (6) and these have been associated with the appearance and persistence of suicidal thoughts and behavior (7).

WHO has developed an international project to evaluate the mental health needs of university students entitled, *World Mental Health International College Student project* [WMH-ICS] (8). Several of the relevant studies within this project have approached the problem of suicide in university students. In this way, Mortier et al. (9) conducted a meta-analysis about the prevalence of thoughts and suicidal behavior, and concluded that these are common in university students worldwide. In Spain, the Project UNIVERSAL forms part of the initiative WMH-ICS. This study covered first year Spanish students in 5 different universities (10). The results of this investigation showed a high prevalence of suicidal ideation (9.9%) in college students (11).

Several studies have found that suicidal thoughts and behaviors in the young are associated with the following distal and proximal risk and protective factors: childhood adversities, positive relationships in childhood, mental disorders, resilience and coping skills, family and social support and recent stressful life experiences (11–19).

On the other hand it is pertinent to state that the COVID-19 pandemic which the world has been living through for over a year has resulted in a common stressor on the general population and has had a great psychological and social impact on university students (20–23), an increase in ideation and suicidal behavior has been observed in this group (24, 25). In addition, it is fundamentally necessary to continue to identify the main risk factors and protection from suicidal behavior in order to prevent the development of more serious and even lethal behaviors. As far as we know, the investigation into suicidal behavior in the Spanish university population is scarce and no such study has been undertaken in the University of Malaga. For these reasons, the objectives of our study were to measure the prevalence of suicidal behavior in a sample of Malaga University students, in the context of the COVID-19 pandemic, and analyze the possible relationship with sociodemographic and academic variables, psychological distress, resilience, family and social support, and the subjective impact of the COVID-19 pandemic.

## Methodology

### Design

An observational and cross-sectional study covering all University of Malaga (UMA) was conducted. An online questionnaire was developed through the web platform “Limesurvey.” The period of time in which to complete the questionnaire was from 3rd March 2021 until 5th April 2021. The information sought in the questionnaire made reference to the previous 6 months with the aim of covering the period from the beginning of the course in September 2020 to the time of data collection. At the beginning of the survey, all students were asked, *via* a survey question, whether they accepted informed consent to participate in the survey. In case the student refused consent, the survey was automatically terminated.

### Participants

According to the UMA website, the number of enrolled students in September 2020 was 36,498, of which 16,501 (45.2%) were men and 19,997 (54.8%) were women. To obtain the size of the sample, the formula for finite populations was used (26). A sampling error of 3%, a confidence level of 95% and a variance of 0.25 were established for a known sample of 36,498 university students. Following the formula for finite populations, the number of subjects necessary to extrapolate the results for the whole population was 1,036.

The strategy for recruitment followed two paths. Firstly, each Dean of Faculty was invited to participate through the University Service of Diffusion by the coordinators of the study. Secondly, the strategy of “snowball sampling” was used between lecturers, colleagues, students, family members and friends in order to reach the maximum numbers of volunteers, as well as the use of social networks for the distribution of the questionnaire. This study was approved by the Ethics Committee for Experimentation of the University of Malaga (CEUMA: 6-2021-H).

The criteria for inclusion were: (a) participants between 18 to 30-years-old; (b) studying in the 2020/2021 course in the University of Malaga; (c) acceptance of informed consent.

### Variables

#### Suicidal behavior

To measure suicidal behavior we used, on the one hand, The Self-Injurious Thoughts and Behaviors Interview [SITBI, (27)] based on the three-step theory of suicide (28) and adapted for Spanish speakers by García-Nieto et al. (29), and on the other hand, the Columbia Suicide Severity Rating Scale [C-SSR, (30)], adapted for Spanish speakers by Al-Halabí et al. (31). The reliability retest of the SITBI items proved sufficient for the Spanish version: *k* = 0.65–1.00 (27, 29), and the internal consistency of the C-SSRS obtained was α = 0.53 (31). Following Blasco et al. (10), items referring to death wishes, suicidal ideation, thoughts of suicide plans, suicide attempts, and self-inflicted injuries throughout life and in the previous 6 months were selected.

For the analysis of associated factors, the variable of result “*risk of suicide”* defined as the sum of all suicidal behavior present in the last 6 months, with the total score between 0 y 5, was used. Including different levels of suicidal behavior in a total score allows those people who present some type of suicide risk to be identified.

According to Nock et al. (27): “the SITBI is a structured interview with 169 items in five modules that assesses the presence, frequency, and characteristics of five types of Self-Injurious Thoughts and Behaviors (SITB): (a) suicidal ideation, (b) suicide plans, (c) suicide gestures, (d) suicide attempts, and (e) nonsuicidal self-injury.” In our study we use only the questions about the presence of this type of behaviors (yes or no). Thus, our outcome variable “suicide risk” is based on the sum of the dichotomous responses (yes/no) of both the SITBI and the C-SSR with respect to each type of suicidal behavior.

#### Psychological distress

The General Health Questionnaire, 12 items version [GHQ-12, (32)], adapted to Spanish by Sánchez-López and Dresch (33), which detects the possible presence of minor mental disorders in the general population. The response options comprise a Likert type scale with 4 alternatives (0 to 3) with a total score of between 0 and 36. A score of 12 or more indicates the presence of psychological distress and a higher score, a more serious degree of distress (34). The GHQ-12 provides a good reliability in the Spanish sample (*α* = 0.76) (33). In the present study, the internal consistency was *α* = 0.89.

#### Resilience

The Brief Resilience Scale [BRS, (35)], was used and adapted to Spanish by Rodríguez-Rey et al. (36), which evaluates unidimensionally the construct of resilience, understood as the capacity of people to recover from life experiences which cause disruptions. There are 6 Likert type items with 5 response options, the scores for which are between 1 and 5. Three items are reverse-corrected and the total score is divided by 6, to obtain a total score of between 0 and 5. The score is interpreted as follows: degree of low resilience (< 3), normal (3–4.30) or high (> 4.30) (37). La BRS presents a good internal consistency (*α* = 0.83) (36). In this present study the internal consistency was *α* = 0.82.

#### Family and social support

The Family and Friends Scale (38) measures the social support perceived through relations with family and friends. It comprises 7 Likert type items with 3 response options with a maximum score of 21. The higher the score the greater the perceived social and family support. According to Bellón et al. (39), the Spanish version provides a good internal consistency index (*α* = 0.88). In this study, the internal consistency was *α* = 0.87.

#### Subjective impact of the COVID-19 pandemic

A multiple choice question was included about the impact of the COVID-19 pandemic in the last 6 months, whereby the participant could select more than one of the options given: *“Motivation,” “Learning,” “Social relations,” “Mood,”* and *“None of the above.”* The variable “*COVID-19 Total*” was created and defined as the sum of all of the selected responses. The score obtained ranged from 0 (minimum) to 4 (maximum) depending on the subjective impact that each person perceived the pandemic to have had on his or her life. Thus, a higher score would indicate a greater impact in different areas of life perceived by the person who had experienced COVID-19.

#### Sociodemographic characteristics

In order to collect the sociodemographic and academic information, an *ad hoc* questionnaire was composed. On the one hand, it included questions about age, gender, nationality, having or not a partner, having or not a job, paternal and maternal education level, living in Malaga or elsewhere, moving out of previous home to undertake studies, the number of flatmates, and the type of relationship with the flatmates with whom they are currently living. On the other hand, questions were asked about the level of the course studies, the academic area, dedication and the modality of classes. In addition the participants studying for a degree also indicated the subject they were studying, the year they were in, the grade needed for access to the university and the preferred position of the degree they were studying.

A description of sociodemographic and academic variables included in the study can be found in Supplementary Table S1.

### Analysis of the data

Firstly, various descriptive analyses were undertaken of the collected variables (frequencies, percentages, means, and standard deviations).

The rates of prevalence were calculated for each type of suicidal behavior, with a confidence interval (CI) of 95%.

Finally, various univariate and multivariate statistical analysis were compiled. Pearson’s correlations, and Student’s t-tests were used together with ANOVA for independent samples to analyze the association between our variable “risk of suicide” and the rest of the independent variables. A multiple linear regression was conducted to verify those variables associated significantly with risk of suicide. In addition, in order to check if the resilience and family and social support mediate or moderate the effect of psychological distress on the risk of suicide, two analyses of mediation and two of moderation were carried out through a bootstrapping process, based on 10,000 bootstrap samples, (40), with PROCESS v.3 for SPSS (41). The models of mediation and moderation were employed where the psychological distress was considered as an independent variable, the resilience and family and social support as mediators and moderators, and the risk of suicide as a dependent variable. The confidence interval was analyzed for the indirect effect and if the interval did not include zero, this indicated an indirect statistically significant effect with *p* < 0.05 (42).

## Results

### Participants

**Figure 1:**
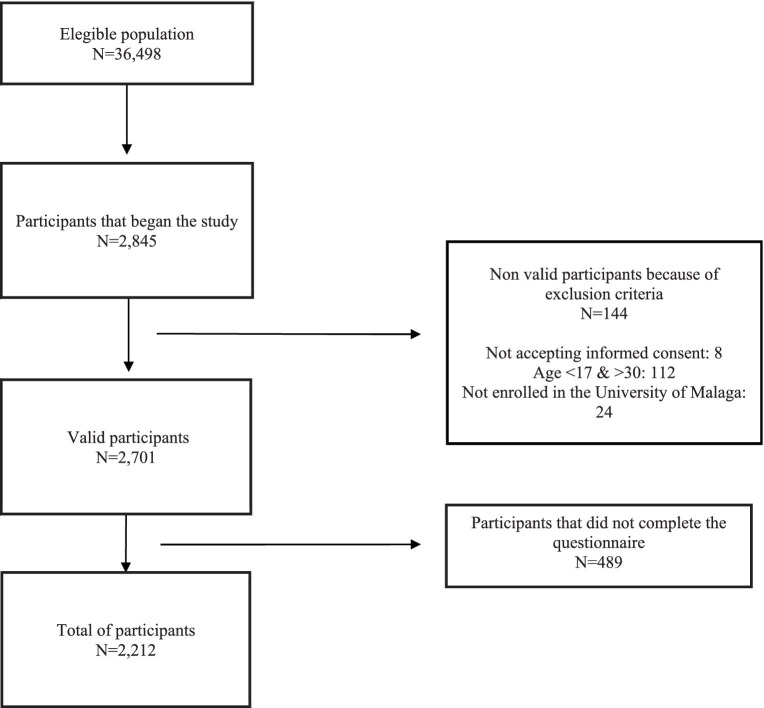
Flow chart of the eligible population.

A total of 2,845 participants completed the questionnaire, 144 were excluded due to the criteria of inclusion and exclusion, of which 8 participants refused to participate and refuse the informed consent. Of the remaining 2,701 participants, 489 questionnaires were not completed until the end and were therefore excluded, thus leaving a total of 2,212 participants (Figure 1).

With regard to gender, there was an unbalanced distribution of the sample with a greater representation of females vs. males (69.6% vs. 30.4%). The average age of the total sample was 21.28 years of age (*SD* = 2.51). The majority of the sample was of Spanish nationality (94.9%), they were exclusively to studying (88.5%), and more than half of their parents did not have a university education (fathers: 71.4%; mothers: 66.2%). With regard to academic information, 92.8% were studying for a university degree and the rest were Masters and PhD students. Most of the participants (29.8%) were studying Social Sciences and Law and were in their second or third academic year (23.8 and 23%, respectively; Table 1).

**Table 1:** Sociodemographic and academic variables.

	Total	Men	Women
	*n* = 2,212 (100%)	*n* = 672 (30.4%)	*n* = 1,540 (69.6%)
*Sociodemographic data*			
	*M (DT)*	*M (DT)*	*M (DT)*
*Age*	21.28 (2.51)	21.58	21.15 (2.41)
	*n (%)*	*n (%)*	*n (%)*
*Nationality*			
Spanish	2,099 (94.9)	648 (96.4)	1,451 (94.2)
Other	113 (5.1)	24 (3.6)	89 (5.8)
*Stable partner*			
Yes	1,054 (47.6)	255 (37.9)	799 (51.9)
No	1,158 (52.4)	417 (62.1)	741 (48.1)
*Have a job*			
Yes	254 (11.5)	84 (12.5)	170 (11)
No	1,958 (88.5)	588 (87.5)	1,370 (89)
*Paternal educational level*			
No University studies	1,580 (71.4)	446 (66.4)	1,134 (73.6)
University studies	632 (28.6)	226 (33.6)	406 (26.4)
*Maternal educational level*			
No University studies	1,465 (66.2)	428 (63.7)	1,037 (67.3)
University studies	747 (33.8)	244 (36.3)	503 (32.7)
*Resident in Malaga (Capital)*			
Yes	1,196 (54.1)	383 (57)	813 (52.8)
No	1,016 (45.9)	289 (43)	727 (47.2)
*Change of residence*			
Yes	894 (40.4)	266 (39.6)	628 (40.8)
No	1,318 (59.6)	406 (60.4)	912 (59.2)
*Number of flatmates*			
1	75 (3.4)	27 (4)	48 (3.1)
2–4	1,889 (85.4)	573 (85.3)	1,316 (85.5)
>4	248 (11.2)	72 (10.7)	176 (11.4)
*Types of flatmates*			
Alone	61 (2.8)	24 (3.6)	37 (2.4)
Family	1,487 (67.2)	450 (67)	1,037 (67.3)
Others	664 (30)	198 (29.5)	466 (30.3)
Academic data			
*Present studies*			
Undergarduate degree	2,052 (92.8)	615 (91.6)	1,437 (93.3)
Master	135 (6.1)	52 (7.7)	83 (5.4)
Doctorate	25 (1.1)	5 (0.7)	20 (1.3)
*Academic field*			
Art y humanities	341 (15.4)	60 (8.9)	281 (18.3)
Social sciences and law	659 (29.8)	161 (24)	498 (32.4)
Health sciences	453 (20.5)	92 (13.7)	361 (23.4)
Sciences	291 (13.2)	98 (14.6)	193 (12.5)
Engineering and architecture	468 (21.2)	261 (38.8)	207 (13.4)
*Academic year*			
First	391 (17.7)	112 (16.7)	279 (18.1)
Second	526 (23.8)	158 (23.5)	368 (23.9)
Third	509 (23)	143 (21.3)	366 (23.8)
Fourth	456 (20.6)	134 (19.9)	322 (20.9)
Fifth	110 (5)	41 (6.1)	69 (4.5)
Sixth	60 (2.7)	27 (4)	33 (2.1)
N/A	160 (7.2)	57 (8.5)	103 (6.7)
*Dedication to study*			
Full time	1,982 (89.6)	600 (89.3)	1,382 (89.7)
Part time	230 (10.4)	72 (10.7)	158 (10.3)
*Modality of classes*			
Presential	23 (1.1)	9 (1.3)	14 (0.9)
Semi-presential	441 (19.9)	159 (23.7)	282 (18.3)
Online	1,723 (77.9)	499 (74.3)	1,224 (79.5)
N/A	25 (1.1)	5 (0.7)	20 (1.3)
	*M (DT)*	*M (DT)*	*M (DT)*
*Grade for University Entry* ^a^	10.07 (1.96)	10.04 (2.04)	10.09 (1.92)
*Preferred position of the degree*	1.4 (1.33)	1.36 (0.95)	1.41 (1.46)

a Range: 0–14.

89.6% of the sample had experienced psychological distress in the last 6 months. Resilience showed a score of 2.83, which indicates a low ability to return to normality after suffering a stressful life event. The participants gained a high score in perceived family and social support with an average of 19.32 out of 21. With respect to the impact of the COVID-19 pandemic, 97.9% indicated that some aspect of their life had been affected by this (Table 2).

**Table 2:** Psychosocial variables and suicidal behavior.

	Total	Men	Women
	*n* = 2,212 (100%)	*n* = 672 (30.4%)	*n* = 1,540 (69.6%)
	*n (%)*	*n (%)*	*n (%)*
*Psychologic distress* *^a^*			
*Present*	1,983 (89.6)	577 (85.9)	1,406 (91.3)
*Absent*	229 (10.4)	95 (14.1)	134 (8.7)
	*M (DT)*	*M (DT)*	*M (DT)*
Resilience^b^	2.83 (0.79)	3.06 (0.77)	2.73 (0.77)
Family/social support^c^	19.32 (2.52)	19.19 (2.62)	19.37 (2.47)
	*n (%)*	*n (%)*	*n (%)*
Death wish			
*Lifetime*			
Yes	1,118 (50.5)	291 (43.3)	827 (53.7)
No	1,094 (49.5)	381 (56.7)	713 (46.3)
*Last 6 months*			
Yes	673 (30.4)	175 (26)	498 (32.3)
No	1,539 (69.6)	497 (74)	1,042 (67.7)
Ideas of suicide			
*Lifetime*			
Yes	744 (33.6)	221 (32.9)	523 (34)
No	1,468 (66.4)	451 (67.1)	1,017 (66)
*Last 6 months*			
Yes	325 (14.7)	105 (15.6)	220 (14.3)
No	1,887 (85.3)	567 (84.4)	1,320 (85.7)
Ideas of suicide plan			
*Lifetime*			
Yes	818 (37)	241 (35.9)	577 (37.5)
No	1,394 (63)	431 (64.1)	963 (62.5)
*Last 6 months*			
Yes	300 (13.6)	93 (13.8)	207 (13.4)
No	1,912 (86.4)	579 (86.2)	1,333 (86.6)
Suicide attempt			
*Lifetime*			
Yes	127 (5.7)	19 (2.8)	108 (7)
No	2,085 (94.3)	653 (97.2)	1,432 (93)
*Last 6 months*			
Yes	11 (0.5)	4 (0.6)	7 (0.4)
No	2,201 (99.5)	668 (99.4)	1,533 (99.6)
Intentional self-harm injuries			
*Lifetime*			
Yes	448 (20.3)	91 (13.5)	357 (23.2)
No	1,764 (79.7)	581 (86.5)	1,183 (76.8)
*Last 6 months*			
Yes	111 (5)	25 (3.7)	86 (5.6)
No	2,101 (95)	647 (96.3)	1,454 (94.4)
	*M (DT)*	*M (DT)*	*M (DT)*
Risk of suicide^d^	0.64 (1.1)	0.6 (1.1)	0.7 (1.1)
Subjective impact of COVID-19 pandemic^e^	2.84 (1.17)	2.73 (1.2)	2.89 (1.16)
	*n (%)*	*n (%)*	*n (%)*
Search for professional help			
*Yes*	758 (34.3)	180 (26.8)	578 (37.5)
*No*	1,454 (65.7)	492 (73.2)	962 (62.5)

GHQ-12, General Health Questionnaire-12 items; BRS, Brief Resilience Scale; FFS, Family and Friends Scale; C-SRRS, Columbia-Suicide Severity Rating Scale; SITBI, Self-Injurious Thoughts and Behaviors Interview.

a Cut-off point: >12.

b Ranges: Low (< 3); Medium (3–4.30); High (>4.30).

c Range: 7–21.

d Range: 0–5.

e Range: 0–4.

### Prevalence of suicidal behavior

With regard to the rates of prevalence in the last 6 months, the results obtained were 30.4% (95% *CI*: 28.5–32.3) death wishes; 14.7% (95% *CI*: 13.2–16.2) suicide ideation; 13.6% (95% *CI*: 12.1–15) ideas of suicide plans; 5% (95% *CI*: 4.1–5.9) self-inflicted injuries; and 0.5% (95% *CI*: 0.2–0.8) suicide attempts. The risk of suicide in the sample was low (*M* = 0.64, *SD* = 1.1), ranging from 0 to 5. Considering gender, females present higher prevalence rates in death wishes (32.3%; 95% *CI*: 30–34.7) and self-inflicted injuries (5.6%; 95% *CI*: 4.4–6.7), while males present higher rates in suicidal ideation (15.6%; 95% *CI*: 12.9–18.4), ideas of suicide plans (13.8%; 95% *CI*: 11.2–16.4) and suicide attempts (0.6%; 95% *CI*: 0.01–1.2; Table 2).

### Association between risk of suicide and other variables

As shown in Supplementary Table S2, significant differences were found between people at higher risk of suicide and having or not a job (*t*(2,210) = 2.567, *p* = 0.01, *d* = 0.19), the academic field (*F*(4, 2,221) = 6.983, *p* < 0.001, *Eta^2^* = 0.01) and the search for professional help (*t*(2210) = −9.107, *p* < 0.001, *d* = 0.44), where the highest risk of suicide was found to be in those not having a job (*M* = 0.66, *SD* = 1.11, vs. *M* = 0.048, *SD* = 0.92), those that searched for professional help (*M* = 0.93, *SD* = 1.29, vs. *M* = 0.49, *SD* = 0.94) and those who were studying for a degree in Arts and Humanities (*M* = 0.87*, SD* = 1.24) in comparison with students studying for degrees in Social Sciences and Law (*M* = 0.60*, SD* = 1.05) and Health Sciences (*M* = 0.47*, SD* = 0.96).

A positive correlation was found between risk suicide and psychological distress (*r* = 0.43, *n* = 2,224, *p* < 0.001) and the subjective impact of the COVID-19 pandemic (*r* = 0.05, *n* = 2,212, *p* = 0.013), and a negative correlation with resilience (*r* = −0.29, *n* = 2,224, *p* < 0.001), family and social support (*r* = −0.30, *n* = 2,224, *p* < 0.001) and the grade for access (*r* = −0.07, *n* = 2,049, *p* = 0.002) on the understanding that those people with the greatest risk of suicide display greater psychological distress, a greater subjective impact from the COVID-19 pandemic, lower resilience, lower perceived family and social support, and lower grade of university entry. No significant differences were found in our variable result with regard to gender.

In the multiple linear regression analysis, the gender variable was included due to its wide relationship with suicidal behavior. As shown in Table 3, psychological distress (*β* = 0.34, *p* < 0.001), resilience (*β* = −0.08, *p* < 0.001), family and social support (*β* = −0.18, *p* < 0.001), the search for professional help, (*β* = 0.12, *p* < 0.001) and the impact of the COVID-19 pandemic (*β* = −0.06, *p* = 0.003) would explain 24% of the variance in the risk of suicide (*R^2^* = 0.24, *F*(9, 2,039) = 72,779, *p* < 0.001).

**Table 3:** Linear regression coefficients.

Model	Non standardized coefficients	Standardized coefficients	*t*	*p*	*Tolerance*	*VIF*
	*B*	*β*				
Constant	1.83		6.07	<0.001		
Gender	0.07	0.03	1.4	0.162	0.87	1.15
Have a job	−0.06	−0.02	−0.80	0.422	0.98	1.02
Art and Humanities	0.14	0.04	1.84	0.066	0.62	1.60
Social Sciences and Law	−0.01	−0.004	−0.16	0.870	0.55	1.82
Health Sciences	0.05	0.02	0.76	0.449	0.57	1.75
Sciences	−0.01	−0.004	−0.16	0.874	0.69	1.44
Grade for university entry	−0.01	−0.02	−0.99	0.323	0.96	1.04
Psychological distress	0.05	0.34	14.16	<0.001	0.66	1.52
Resilience	−0.12	−0.08	−3.65	<0.001	0.73	1.36
Familiar/social support	−0.08	−0.18	−8.88	<0.001	0.90	1.11
Search for professional help	0.29	0.12	6.30	<0.001	0.95	1.05
Subjective impact of COVID-19 pandemic	−06	−0.06	−2.93	0.003	0.92	1.09

Dependent variable: suicide risk.

With regard to the simple mediation analysis, the first of these revealed that the total effect of psychological distress on the risk of suicide was significant (*B* = 0.06, *S.E.* = 0.003, *p* < 0.001). In the same way, it also shown that the direct effect of psychological distress on the risk of suicide was significant (*B* = 0.05, *S.E.* = 0.003, *p* < 0.001). Finally, an indirect effect of psychological distress on suicide risk was also found through the family and social support (*B* = 0.01, *S.E.* = 0.001, *CI* [0.006, 0.011]; Supplementary Figure S1).

The second simple mediation analysis showed an indirect effect of psychological distress on the risk of suicide, through resilience (*B* = 0.01, *S.E.* = 0.002, *CI* [0.005, 0.012]; Supplementary Figure S2).

**Figure 2:**
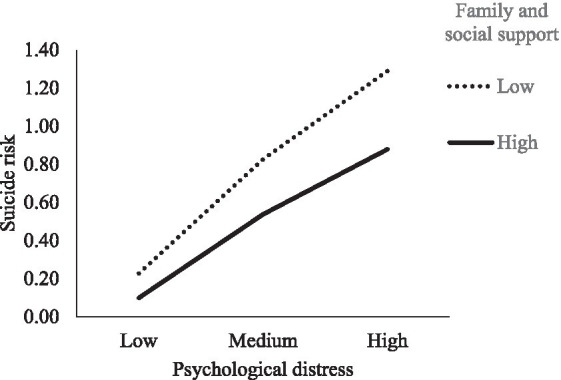
Moderation of family and social support on the relationship between psychological distress and suicide risk.

On the other hand, the first moderation analysis showed that family and social support moderated the relationship between psychological distress and the risk of suicide (*B* = <−0.01, *S.E.* = 0.001, *p* < 0.001, *CI* [−0.007, −0.002]). As seen in Figure 2, an effect of psychological distress on the risk of suicide was also observed in both cases of low levels of family and social support (*B* = 0.07, *S.E.* = 0.004, *CI* [0.059, 0.074]), and high levels (*B* = 0.05, *S.E.* = 0.003, *CI* [0.042, 0.055]). These results reveal that family and social support reduce the relationship between psychological distress and the risk of suicide. That is to say, even though psychological distress increases the risk of suicide, it is reduced through the influence of family and social support.

**Figure 3:**
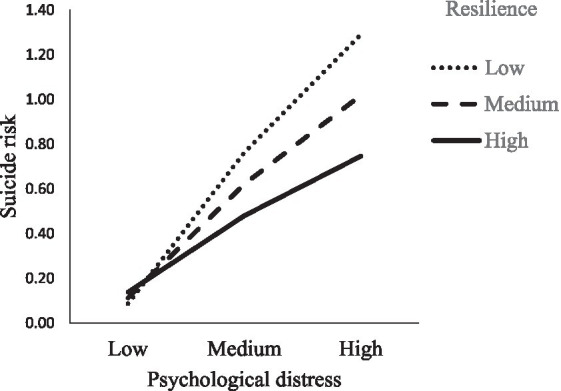
Moderation of resilience on the relationship between psychological distress and suicide risk.

Finally, the second moderation analysis revealed that resilience moderated the relationship between psychological distress and the risk of suicide (*B* = −0.02, *S.E.* = 0.003, *p* < 0.001, *CI* [−0.029, −0.016]). Thus a reducing effect of resilience on the relationship of psychological distress and the risk of suicide on the low levels of resilience (*B* = 0.08, *S.E.* = 0.004, *CI* [0.067, 0.084]), medium (*B* = 0.57, *S.E.* = 0.003, *CI* [0.050, 0.063]) and high levels (*B* = 0.04, *S.E.* = 0.004, *CI* [0.030, 0.046]; Figure 3).

## Discussion

Our study, as far as we know, is the first to provide data on the prevalence of suicidal behavior in students from the University of Malaga (Spain). We highlight that psychological distress is a risk factor for suicidal behavior, while resilience and family and social support are linked to a lower risk of suicide.

We can observe that the percentage of women who responded to the survey (69.6%) is much higher than the percentage of women enrolled in the university (54.8%). This result is in line with Noy (43) indicating that women are more likely to participate in this type of survey, as well as being more likely to seek help form mental health problems than men (44–46).

The results of our study show that the prevalence of suicidal ideation, ideas of suicide plans and suicide attempts lifetime, in students from the University of Malaga, is higher than those of other studies carried out in university students where results obtained were 6–32.7% for ideation, 2.3–17.5% for ideas of plans and 1.4–4.3% for suicide attempts (9, 47, 48). In addition, the prevalence reported in our study is much higher than that found in a systematic and meta-analysis of European studies in the general population (49). However, the prevalence in the last 6 months are, in general, lower in our study than others (7, 9, 11, 47, 48). This may be due to the fact that the other studies measured suicidal behavior in the last 12 months while our study covered 6 months which was the period from the beginning of the beginning of the academic year until the collection of data (6 months later).

Several studies found that the presence or appearance of disorders such as depression, anxiety and, substance use during adolescence or adulthood, is associated with a greater risk of the onset and persistence of suicidal thoughts and behavior (50, 51). In our study, the risk of suicide is associated with psychological distress. The results are consistent with those of Blasco et al. (12), where the presence of a mood disorder is the principal risk factor associated with the appearance and persistence of suicidal behavior in students. On the other hand, our results suggest that resilience together with family and social support provide a protective effect against the risk of suicide. These findings are consistent with those found in other studies that show that resilience as much as positive relationships with peers and family members are associated with a lower probability of the appearance and persistence of suicidal behavior in universities (11, 12, 52) and in the general population as a whole (53).

Concerning the impact of the COVID-19 pandemic, being young, that is, those aged between 18 and 24, are a prediction factor of suicidal thoughts presence (54, 55). In this way, various studies found an increase in thoughts of self-harm and suicide among students during periods of self-isolation (24, 56). These results are consistent with our study which shows a relationship between the risk of suicide and the effect that the COVID-19 pandemic has had on the suicide behavior of university students.

Finally, it would appear that university students who look for professional help are at the most risk of suicide, and as a consequence of this, the services that offer psychological and psychiatric help to this population should take into account both suicidal behavior in both their evaluations and interventions.

Among the strengths of our study, we highlight that, as far as we know, this is the only study which measures suicidal behavior of students at the University of Malaga (Spain) during the COVID-19 pandemic. Another strength is the high response to our questionnaire, resulting in a representative sample of the study population. In addition, our study is sustained by the data of the international Project WMH-ICS and in the UNIVERSAL study in Spain, thus expanding the scope of the sample to all university students.

Nevertheless, our results are not exempt from limitations and must be interpreted with a certain caution. Firstly, even though the situation of COVID-19 brings a unique value to the study, this in itself makes it extremely difficult to compare results with other studies as the impact the pandemic has had on the general population may be giving higher scores. On the other hand, our study is limited in time as we have collected data only from the last 6 months, which may make comparison with other studies difficult. Moreover, participants were a non-probabilistic sample of university students from different faculties, for this reason the validity of our findings may be somewhat hindered by this type of sampling approach (57). In addition, we have used self-report instruments to assess mental health variables, however, as other studies have indicated (21, 52, 58, 59) these types of instruments have been very important in collecting information during the COVID-19 pandemic in the university population and, specifically, the study by Núñez et al. (60) demonstrates that the self-report version of the C-SSR is a useful instrument for assessing suicidal ideation in adolescents, as well as distinguishing between subjects at low and high risk of suicide. Finally, it is important to point out that few variables were analyzed, and omitted some important ones relating to suicide such as substance use, stressful life events or major mental disorders.

In conclusion, our study show links between suicide risk and psychological distress, resilience and perceived family and social support in college students which indicate that those who present any anxiety/depression disorder or symptoms, with a low ability to return to normality after suffering a stressful life event and who feel they have a poor or no relationship with their relatives, are at a greater risk of suicide. One must also take into account that the context of this study was the COVID-19 pandemic and this may influence the results, aggravating them in comparison with previous studies. For this reason, and given the importance of the matter discussed here, it would be necessary to undertake a future study outside of the pandemic, as well as periodic studies to measure the risk of suicide.

## Data availability statement

The original contributions presented in the study are included in the article/Supplementary material, further inquiries can be directed to the corresponding author.

## Ethics statement

The study was approved by the Ethics Committee for Experimentation of the University of Malaga (CEUMA: 6-2021-H). Written informed consent to participate in this study was provided by all students before completing the survey.

## Author contributions

JR-M, JP-B, JO, and BM-K contributed to the study’s conception and design, and performed the material preparation, data collection, and analysis. JR-M and JP-B was written the first draft of the manuscript. All authors commented on previous versions of the manuscript. All authors read and approved the final manuscript.

## Conflict of interest

The authors declare that the research was conducted in the absence of any commercial or financial relationships that could be construed as a potential conflict of interest.

## Publisher’s note

All claims expressed in this article are solely those of the authors and do not necessarily represent those of their affiliated organizations, or those of the publisher, the editors and the reviewers. Any product that may be evaluated in this article, or claim that may be made by its manufacturer, is not guaranteed or endorsed by the publisher.
