# Supplementary material for: Non-lethal suicidal behavior in university students of Spain during COVID-19
Source: Front Psychiatry. 2023 Jul 18;14:1155171. doi: 10.3389/fpsyt.2023.1155171 (PMC10390698; doi:10.3389/fpsyt.2023.1155171)
Supplement: Supplementary file 1 [file Data_Sheet_1.docx]

**Supplementary Material**

**Tabla S1.** Description of the sociodemographic and academic variables collected for the study.

| **Variable** | **Description** |
| --- | --- |
| ***Sociodemographic variables*** |  |
| Gender | Identities with which people identify |
| Age | Number of years |
| Nationality | The status of belonging to a particular nation |
| Stable Partner | Having or not partner |
| Have a job | Having or not an actual job |
| Paternal and maternal education level | The highest educational degree they've obtained |
| Residence | The place where a person live |
| Change of residence | Moving to another address |
| Number of flatmates | Number of people with whom you share residence |
| Type of flatmates | Relationship with persons sharing residence |
| ***Academic variables*** |  |
| Present studies | Studies currently being pursued |
| Academic field | A subdivision of knowledge that is taught and researched at the college or university level |
| Academic year | Current academic year from first to sixth |
| Dedication to study | Time dedicated to studies (full time, part time...) |
| Modality of classes | Type of classes (presence, online…) |
| Grade for university entry | Average grade point average for university entrance |
| Preferred position of the degree | Selection of the career that a person wants to study in order of preference |

**Table S2. Correlation analyses of sociodemographic, academic, psychosocial, help-seeking and COVID-19 subjective impact with risk of suicide**

|  | Bivariates | |
| --- | --- | --- |
|  | Stadistics | *P* |
| **Sociodemographic variables** |  |  |
| *Age* | -.01^a^ | .604 |
| *Gender* | -1.24^b^ | .214 |
| *Nacionality* | -1.36^b^ | .172 |
| *Stable partner* | 1.38^b^ | .166 |
| *Employed* | 2.56^b^ | .010* |
| *Paternal Educational Level* | 1.36^b^ | 1.73 |
| *Maternal Educational Level* | -.14^b^ | .887 |
| *Resident in Malaga (Capital)* | 1^b^ | .315 |
| *Change of Residence* | .19^b^ | .846 |
| *Number of flatmates* | 1.665^c^ | .190 |
| *Type of flatmates* | .100^c^ | .904 |
| **Academic variables** |  |  |
| *Present studies* | 2.372^c^ | .094 |
| *Academic field* | 6.983^c^ | <.000*** |
| *Academic year* | 1.569^c^ | .165 |
| *Dedication to study* | -1.55^b^ | .121 |
| *Modality of clases* | .218^c^ | .804 |
| *Grade of university entry* | -.07^a^ | .002** |
| *Preferred position of the degree* | .00^a^ | .892 |
| **Psychosocial variables** |  |  |
| *Psychological distress* | .42^a^ | <.000*** |
| *Resilience* | -.29^a^ | <.000*** |
| *Familiar/social support* | -.29^a^ | <.000*** |
| **Search for professional help** | -9.10^b^ | <.000*** |
| **Subjective impact of COVID-19 pandemic** | .05^a^ | .013* |

^a^ Pearson´s r*;* ^b^ Student´s t-test*; ^c^ F* ANOVA

**p*<.05; ***p*<.01; ****p*<.001

**Figure S1. Mediation model of family and social support**


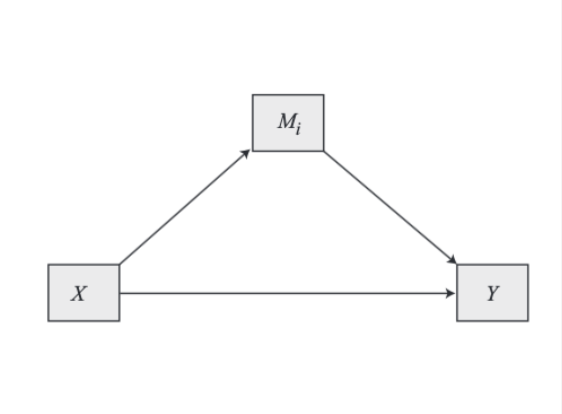


Psychological distress

Suicide risk

X→M→Y

Indirect effect (ab)

*B* = 0.01, *S.E.*= 0.001, 95% *CI* [0.006, 0.011]

C’ = 0.06^**^

C = 0.05^**^

Family and social support

a = -0.09^**^

b= -0.08^**^

*c = total effect of X on Y; c’ = direct effect of X on Y; a = effect of X on M; b = effect of M on Y.* **p* < .05; ***p* < .01

**Figure S2. Mediation model of resilience**


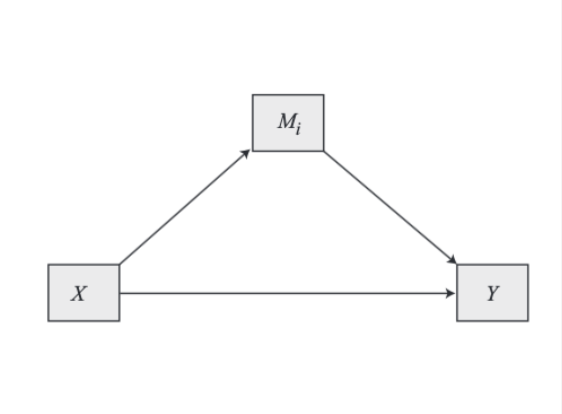


Psychological distress

Suicide risk

Resilience

X→M→Y

Indirect effect (ab)

*B* = 0.01, *S.E.*= 0.002, 95% *CI* [0.005, 0.012]

a = -0.05^**^

C’ = 0.06^**^

C = 0.05^**^

b= -0.17^**^

*c = total effect of X on Y; c’ = direct effect of X on Y; a = effect of X on M; b = effect of M on Y.* **p* < .05; ***p* < .01
